# We Have a Lot to Do: Lack of Sexual Protection and Information—Results of the German-Language Online Survey “Let's Talk About Chemsex”

**DOI:** 10.3389/fpsyt.2021.690242

**Published:** 2021-05-31

**Authors:** Cornelia Rosenberger, Marcus Gertzen, Moritz Strasburger, Johanna Schwarz, Solveig Gernun, Andrea Rabenstein, Eva Lermer, Tobias Rüther

**Affiliations:** ^1^Department of Addiction Medicine, kbo-Isar-Amper-Klinikum Munich East, Haar, Germany; ^2^Psychotherapy and Psychosomatics, Medical Faculty, Bezirkskrankenhaus Augsburg, University of Augsburg, Augsburg, Germany; ^3^Department of Psychiatry, Ludwig-Maximilians-University Hospital Munich, Munich, Germany; ^4^FOM University of Applied Sciences for Economics and Management, Munich, Germany; ^5^LMU Center for Leadership and People Management, Ludwig-Maximilians-University, Munich, Germany

**Keywords:** chemsex, MSM, 4 chems, WHO-5, sexual motivated substance use

## Abstract

**Background:** The prevalence of chemsex and sexualized substance use is increasing in several European countries, particularly among men who have sex with men. In this subgroup, illegal substance use is associated with increased sexual risk behavior, which can result in severe physical and psychological impairments. The present study examined the incidence and prevalence of chemsex in German-speaking countries.

**Methods:** To further describe the high-risk group of Chemsex users, participants (*N* = 429) were asked about their psychotropic substance use, sexual and health-related behavior, health status, and socio-demographic information by using an online questionnaire. Whether Chemsex has negative effects on well-being was measured with the WHO well-being index. Of additional interest was how informed Chemsex users are about the topic and what needs are placed on the practitioners. The online questionnaire consisted of 105 items, and data was collected from March to May 2019. Thousand forty seven datasets were saved with a dropout rate of 59%, 123 completed questionnaires fulfilled the criteria for chemsex users (*n* =123).

**Results:** There were no significant differences in well-being between chemsex users and non-users. All participants protected themselves against sexually transmitted diseases irregularly or not at all. The majority of chemsex users reported intermittently using illegal substances (ketamine, methamphetamine, mephedrone, γ-butyrolactone/γ-hydroxy butyric acid). They viewed their sexual and substance use behavior as problematic, but few showed motivation for behavior change. Chemsex users clearly expressed a need for more information and advice centers.

**Conclusion:** The results provide information on chemsex users that can be used for the future development of a therapy manual and thus contribute to improving health care for this group. The prevalence of chemsex is increasing and urgently needs more research to protect clients from health impairments and stigmatization.

## Introduction

The use of mind-altering substances is a well-known social phenomenon, and population-wide substance use remains a matter of concern. Approximately 269 million adults worldwide used illicit drugs at least once in 2018 (1), with 29% of those aged 15–64 estimated to be from the European Union (2).

In the 15 to 64 age group, problematic use is described in this context; of the 11.3 million i. v. users, about half were infected with hepatitis C and about 12.6% with HIV (3). According to the United Nations Office on Drugs and Crime, around 35 million people suffer from health disorders caused by drug use and require treatment services (1).

Two specific types of substance use are chemsex and sexual motivated substance use, whereby the former describes the extent of and motivation for substance use more explicitly than the latter (4). The literature differentiates between the 2 terms because of the higher risk potential of chemsex (5). Chemsex is used to initiate sexual acts and increase or disinhibit the pleasure, duration, and intensity of the sexual experience (4, 6, 7). It is also referred to as “party and play” (8, 9).

Chemsex users take one or more illegal psychoactive substances to influence sexual perception and behavior and enable long-lasting sex sessions, sometimes over several days, or sex with several partners simultaneously (5, 6, 8, 10, 11). Four illegal psychoactive substances are mainly used for chemsex: methamphetamine, mephedrone, ketamine and γ-butyrolactone (GBL)/γ-hydroxin butyric acid (GHB) (4, 9). These substances, also referred to as the “4 chems” (5, 9), are mostly consumed orally or nasally, although intravenous use does occur (known as slam sex) (12). Chemsex substances differ from the “party drugs” used for recreation, although these recreational activities may include sexual activities; the preferred party drugs include ecstasy, amphetamines, and cocaine (13–15). The four chems have an increased addiction potential and can also cause lasting damage. Ketamine use can lead to near-death experiences and trigger drug-induced psychosis (16). Overdoses of mephedrone may cause thought disorders, anxiety states, panic attacks, acoustic hallucinations, palpitations, nausea, and vomiting (17–19). The negative effects of methamphetamine include motor restlessness, aggressiveness, panic attacks, concentration disorders, and long-term effects on the brain. GHB/GBL overdose can lead to a loss of consciousness or even respiratory depression (20–22). In addition, there is the danger of unconscious mixed use, which is caused by contaminated substances. For example, the case report by Pichini et al. described how two people consciously used GHB in a sexual context, but the substance was unknowingly mixed with sildenafil, which in turn led to physical discomfort and even a visit to the emergency room (23). Besides these negative effects of drug use among chemsex users, other problematic aspects include criminalization through illegal procurement of the substances and the risk of social decline (20).

Generally, substance use is higher among lesbian, gay, bisexual, and transgender (LGBTQ) people than among purely heterosexual people (24), partly because of sexual substance use (25). Chemsex occurs predominantly among men who have sex with men (MSM) (6). Peer groups have been shown to have a stronger influence on sexual behavior than family members (26), particularly in the MSM community (27). In a qualitative study on the experiences of 30 MSM, the interviewees reported that drug use and in particular chemsex is now perceived as being ubiquitous in the MSM community (7). Often, sex dates are arranged through mobile dating apps such as Grindr. Before they meet, the parties not only arrange the location but also exchange information including the type of substance use, HIV status, and number of participants. This virtual communication allows like-minded people to meet each other without having to visit bars, clubs, or similar locations and allows them to meet their sexual needs more quickly and effectively (5, 28).

Chemsex is associated with increased health risk behavior, which can lead to increased rates of sexually transmitted diseases (STDs) and even to death from drug intoxication (11, 28–30). MSM are considered to be one of the main risk groups for new infections with STDs such as HIV (31–34). Safer sex practices are often neglected during (group) sex sessions with substance use, and needles may also be shared (35). The disinhibition resulting from substance use means that chemsex users often consume more psychoactive substances than planned, and a lack of knowledge about the dosage can lead to severe intoxications and even to fatal overdoses (12). The European MSM Internet Survey, the first study to examine the extent and spread of chemsex in Germany (35, 36), found a significant association between psychotropic substance use and risky sexual behavior (37). For example, 26% of the study participants in Germany (*n* = 54,387) stated that they had unprotected anal intercourse with men. The study also showed a positive association between positive HIV status and increased risky health behavior, such as promiscuity and unprotected sexual intercourse (35).

In spring 2018, the Ludwig-Maximilians-University Hospital Munich, department of psychiatry opened a special outpatient clinic to provide support to chemsex users, and the clinic is seeing increasing numbers of people. The counseling and treatment of people with sexual motivated substance use is problematic mainly because of the combination of harmful use of psychotropic substances and sexual risk behavior. Compared with data on other psychiatric phenomena, data on the prevalence of chemsex and characteristics of chemsex users is still lacking (6), especially in German-speaking countries. Furthermore to our knowledge, no standardized guidelines or therapy programs exists. Therefore, the present study aimed to better define the group of people that engages in chemsex, obtain data on their use of chemsex and needs for advice and information, and assess their well-being. The specific research questions were as follows:

A. Can demographic variables be used to identify a high-risk group for chemsex?
B. What are the main characteristics of the sexual and health behavior and frequency of use of psychotropic substances, in particular the 4 chems?
C. Does chemsex affect the well-being of users?
D. What do users know and need from clinics on the topic of chemsex?

This project has been approved by the ethical committee by the Ludwig-Maximilians-University Hospital Munich, department of psychiatry and was registered bay the number: 18–819 KB.

## Materials and Methods

### Recruitment and Sample

Participation in this field study was limited to people in German-speaking countries. Persons of all gender and legal age and who had functioning online access could participate in the survey. Due to the exploratory character of this study and the aspects that chemsex users belong to a group that is difficult to identify and locate, the method of snowball sampling was chosen for statistical purposes. Participants were mainly recruited from the LGBTQ community. They were recruited by flyers in the psychiatric outpatient clinic at the Ludwig-Maximilians-University Hospital Munich, department of psychiatry, at regional scientific congresses, and nationwide via AIDS and addiction advice centers, LGBTQ contact and advice centers, specialist hospitals for the treatment of addiction disorders, and relevant bars in large German cities. People on the email distribution list of the Ludwig Maximilian University Munich were also invited to participate. The study was also publicized on social media such as Planet Romeo (a dating app for homosexual and bisexual men), the homepage of the chemsex outpatient Ludwig-Maximilians-University Hospital Munich, department of psychiatry, the Facebook page of the interdisciplinary HIV Center IZAR of the Technical University Munich, and via the personal Facebook and Instagram accounts of the authors. Articles about the study in 2 digital lifestyle magazines for homosexual and bisexual men, *Boner Magazine* and *GLEICHLAUT*, also encouraged readers to participate.

### Assessment

Participants anonymously completed an online questionnaire between March 2 and May 31, 2019. The questionnaire inquired about sociodemographics, sexual and health behavior, frequency of psychotropic substance use, and well-being. It included a list of legal and illegal controlled substances in Germany that was based on the medical guidelines on health care for methamphetamine-related disorders (38). The order, content, and number of items in the questionnaire are shown in Table 1. At the end of the questionnaire, all participants were able to leave personal comments as free text.

**Table 1:** Content of the questionnaire.

**Order**	**Content**	**Number of items**
1.	Age, sex, sexual orientation	3
2.	WHO Well-Being Index (WHO-5)	5
3.	Relationship Scales Questionnaire (RSQ)	30
4.	List of psychotropic substances	23
5.	Number of sexual partners in the past 12 months and sexual behavior	14
6.	Level of sexual activity, chemsex, and attitude toward chemsex	6
7.	Length of time having sex without the influence of alcohol or psychotropic substances	2
8.	Sexual activity under the influence of alcohol	1
9.	Protective behavior with respect to sexually transmitted diseases	7
10.	Health status with respect to sexually transmitted diseases	3
11.	Current status and needs regarding advice centers on chemsex	4
12.	Sociodemographics	7
13.	Seriousness about answering the online questionnaire	1

To assess substance use, participants were asked about their use of 23 psychotropic substances in the past 12 months (options: never, about once every 3 months, about once a month, about once a week, several times a week, daily). Sexual behavior was assessed on a 7-point Likert scale ranging from 1 (strongly disagree) to 7 (strongly agree). Participants used the scale to rate how strongly they agreed that in the past 12 months they had had sex while under the influence of substances, had participated in group sex, or had attended private sex parties. They were also asked how and where they had found sexual partners (eg, “I use online sites to arrange sex, for example, PlanetRomeo, Scruff, Grindr), who they preferred having sex with (e.g., men, women, people they know, or sex workers), and whether and how often their sexual behavior was influenced by illegal substances. Additional questions inquired whether participants saw themselves as chemsex users, viewed their own chemsex behavior as problematic and wanted to change their behavior. The topic of safer sex practices was also assessed with a 7-point Likert scale ranging from 1 (strongly disagree) to 7 (strongly agree); questions concerned the use of condoms, drugs for HIV pre- and post-exposure prophylaxis, and hepatitis A and B immunization status. Participants were also asked to provide information as free text about safer sex practices not included in the list and to specify whether they used any safer sex practices at all. We were also interested to find out whether participants took measures to protect themselves from STDs depending on whether they were having sex inside or outside a partnership.

Study participants were asked when they last had sex while not under the influence of alcohol or illegal substances (response options: in the past week, month, 3 months, 6 months, 12 months, and longer than 12 months). They were also asked whether they drank alcohol before and during sex, which was assessed on a 7-point Likert scale ranging from 1 (never) to 7 (always). To assess participant health status, the questionnaire asked about current or past STDs and the time of the last test for such diseases. Participants were also asked about their knowledge of contact points and advice centers and about current and past psychotherapy and psychiatric treatment. When responding to the questions, participants were asked to consider the past 12 months because the study aimed to obtain an overview of the current epidemiology of chemsex.

The participant's current level of well-being was assessed with the WHO-5 (39). This scale is a brief psychometric questionnaire that uses five items to inquire about subjective well-being in the past 2 weeks (40). The scale has good reliability (Cronbach‘s alpha = 0.92). A sum score of 0 to 13 indicates a low or negative well-being.

### Statistical Analysis

Data were analyzed with R, version 3.3.1 (June 21, 2016). Participants were categorized as chemsex users and non-users on the basis of their response on a 7-point Likert scale ranging from 1 (strongly disagree) to 7 (strongly agree) to the question whether in the past 12 months they had used illegal controlled substances to influence their sexual behavior. Participants who responded with “strongly disagree” on the Likert scale were categorized as non-users; all other participants were categorized as chemsex users, the cut-off score was 2.

Data were tested for normal distribution with the Shapiro-Wilk test (*P* < 0.05) and for homogeneity of variance with the Levene test (*P* =.29). A *t* test was used to calculate whether differences existed in the mean well-being of chemsex users and non-users.

## Results

**Figure 1:**
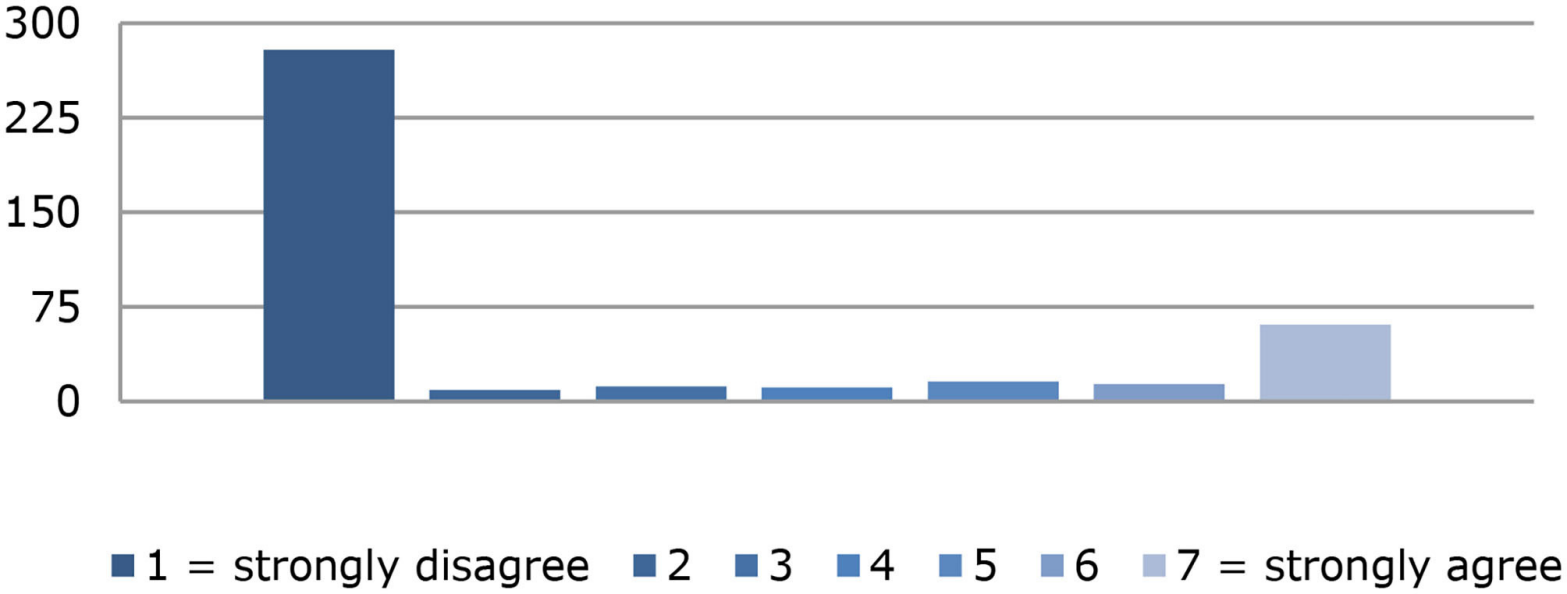
Frequency distribution of the item: “within the last 12 months I had sex under the influence of drugs”.

At the end of data collection, 1,047 data records were available. The first screening showed that 618 participants did not complete the questionnaire, so 59% their data could not be used for statistical hypothesis testing. Among the remaining 429 data sets that were suitable for further analysis, 123 were completed by participants who stated that they had had sex under the influence of illegal substances in the past 12 months [mean (SD) = 5.87 (1.69)], Figure 1 shows the frequency distribution for this item. The mean (SD) age of all participants was 35.00 (13.01) years; and of the chemsex users, 42.97 (11.66) years.

### Sociodemographic Characteristics

Most of the participants described themselves as male, and almost half belonged to the MSM group. The majority of the sample were living in a partnership, had a college degree and were employed.

There were no difference when using the *t* test according to the WHO-5 between the chemsex users and non-users, that is, it did not indicate negative well-being in either group (*P* = 0.58). The mean (SD) well-being score was 17.72 (4.65; median, 18; range, 25) in the group of chemsex users (*n* = 123) and 17.98 in the group of non-users (4.13; median, 19; range, 25). Thus, both groups were above the cut-off score of 13 (see 2.2 above). A Cohen's d of 0.59 resulted in an effect strength of 0.030.

Among the chemsex users, 93% described themselves as male and 7% as female; 1 person in the group of chemsex users described themselves as transsexual. The majority of chemsex users described themselves as gay (72%), followed by bisexual (21%) and heterosexual (7%). Regarding partnerships, 40% stated that they were living with a partner, 15% were married, 40% were single, and 6% were in other types of partnerships (e.g., open relationship). Almost half of the chemsex users (46%) had a college degree, and 41% were employees. More than half (57%) had a monthly net income between €1,000 and €3,500. Table 2 shows the sociodemographic characteristics of the participants.

**Table 2:** Sociodemographic characteristics of participants in an online survey of chemsex use.

**Variable**	**Whole sample *(N* = 429) %**	**Chemsex users (*n* = 123) *n***	**Non-users (*n* = 306) *n***
**Sex**			
Male	64%	93	53%
Female	35%	7	46%
Transsexual	1%	0	1%
Intersexual	0%	0	0%
**Sexual orientation**			
Heterosexual	40%	7%	54%
Gay	40%	72%	27%
Lesbian	1%	0%	1%
Bisexual	19%	21%	18%
**Partnership**			
With a partner	47%	40%	50%
Married	12%	15%	11%
Single	36%	40%	35%
Other	4%	6%	4%
**Highest educational level**			
No school diploma	-	0%	0%
School diploma after grade 9 or 10	4%	7%	3%
Vocational or high school diploma	29%	17%	33%
Completed apprenticeship/vocational training	15%	20%	13%
College degree	46%	46%	46%
PhD	6%	10%	5%
**Employment status**			
Student at school or college/apprentice	33%	8%	43%
Employee	32%	41%	29%
Senior staff	8%	9%	8%
Middle management	6%	11%	4%
Upper/top management	3%	7%	2%
Self-employed	10%	14%	8%
Retired	4%	6%	3%
Not currently employed	1%	2%	0%
Other	3%	2%	3%
**Monthly net income**			
< €1,000	28%	10%	34%
€1,000–€2,000	28%	29%	22%
€2,000–€3,500	24%	28%	25%
€3,500–€5,000	26%	19%	8%
€5,000–€6,500	11%	4%	3%
Over €6,500	4%	9%	3%
State benefits	4%	1%	5%

### Substance Use in the Group of Chemsex Users

**Figure 2:**
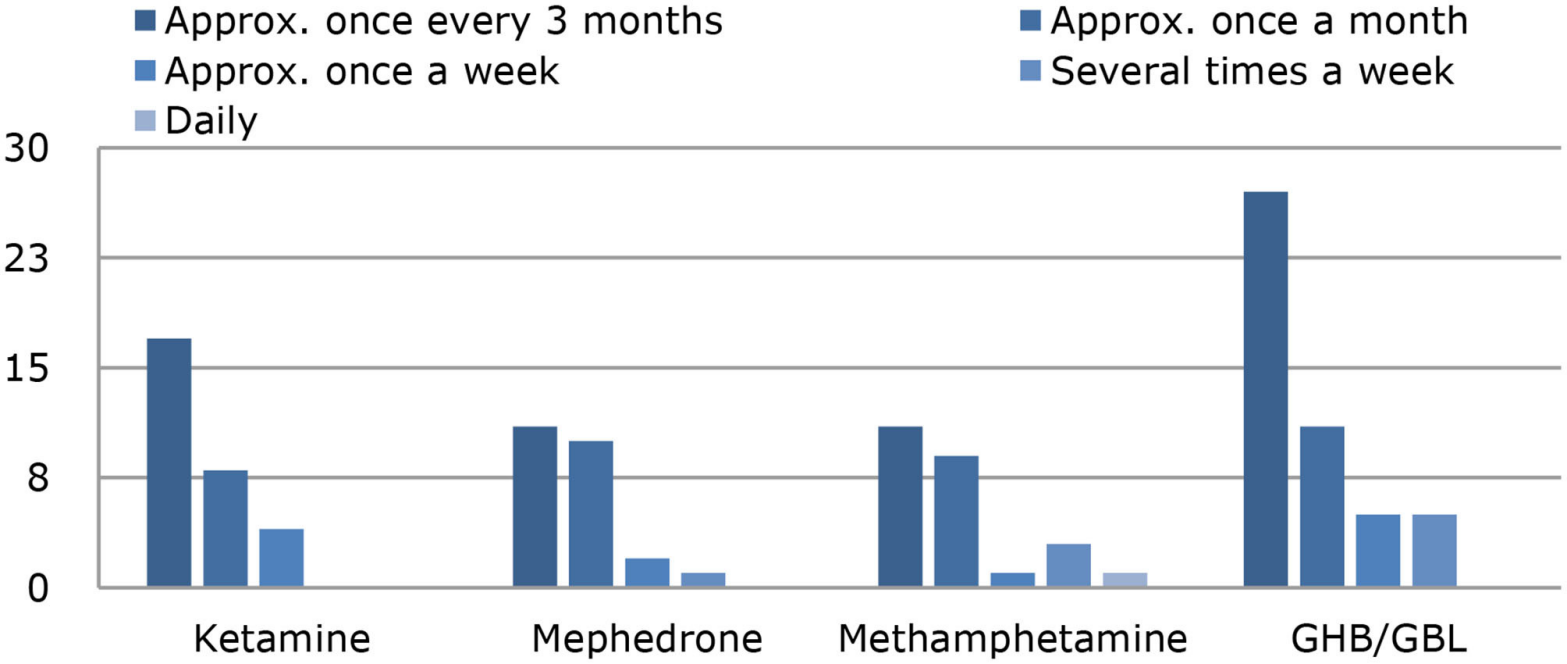
Use of the 4 chems in the group of chemsex users (*n* = 123) in the past 12 month.

Among the chemsex users (*n* = 123), 71% had not used ketamine in the past 12 months, but 14% had used about once every 3 months; 7%, about once a month; and 3% about once a week. None of the chemsex users used ketamine several times a week or daily, and 6% of chemsex users did not provide any information. Regarding methamphetamine, 76% had not used it in the past 12 months, but 9% had used it about once every 3 months; 7%, about once a month; 2%, several times a week; and 1%, daily. Information on methamphetamine use was missing for 4% of the chemsex users. Mephedrone had not been used in the past 12 months by 71% of the chemsex users, but 14% had used it about once every 3 months; 7%, about once a month; and 3%, about once a week. One person used mephedrone several times a week, and 6% did not provide any information. GHB/GBL had not been used in the past 12 months by 58% of the group of chemsex users, but 22% had used it about once every 3 months during this period, 9% about once a month, 4% about once a week, 4% several times a week, and 3% did not provide any information. The current intoxication when answering the questionnaire was not recorded. Use of the 4 chems by the chemsex users is shown in Figure 2.

### Sexual and Health Behavior in the Group of Chemsex Users

**Figure 3:**
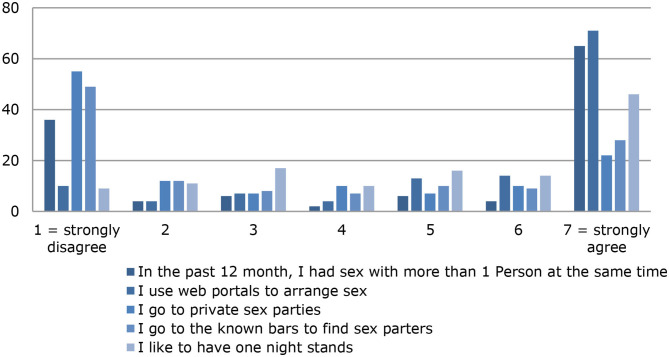
Sexual behavior.

In the past 12 months, 71% [mean (SD) = 4.71 (2.79)] of the chemsex users had sex with more than one person at the same time, and the mean (SD) number of sexual partners in the past 12 months was 24.59 (33.49) (median = 12; range, 0–240). Most people in the group of chemsex users [92%; mean (SD) = 5.70 (1.95)] used web portals such as Grindr or Planet Romeo to arrange sex, and 60% [mean (SD) = 3.46 (2.49)] visited known bars, such as dark rooms or swinger clubs, to find sexual contacts. Just over half of chemsex users [55%; mean (SD), 3.16 (2.41)] stated that they had participated in private sex parties. An overview of sexual behavior in the past 12 months is shown in Figure 3.

**Figure 4:**
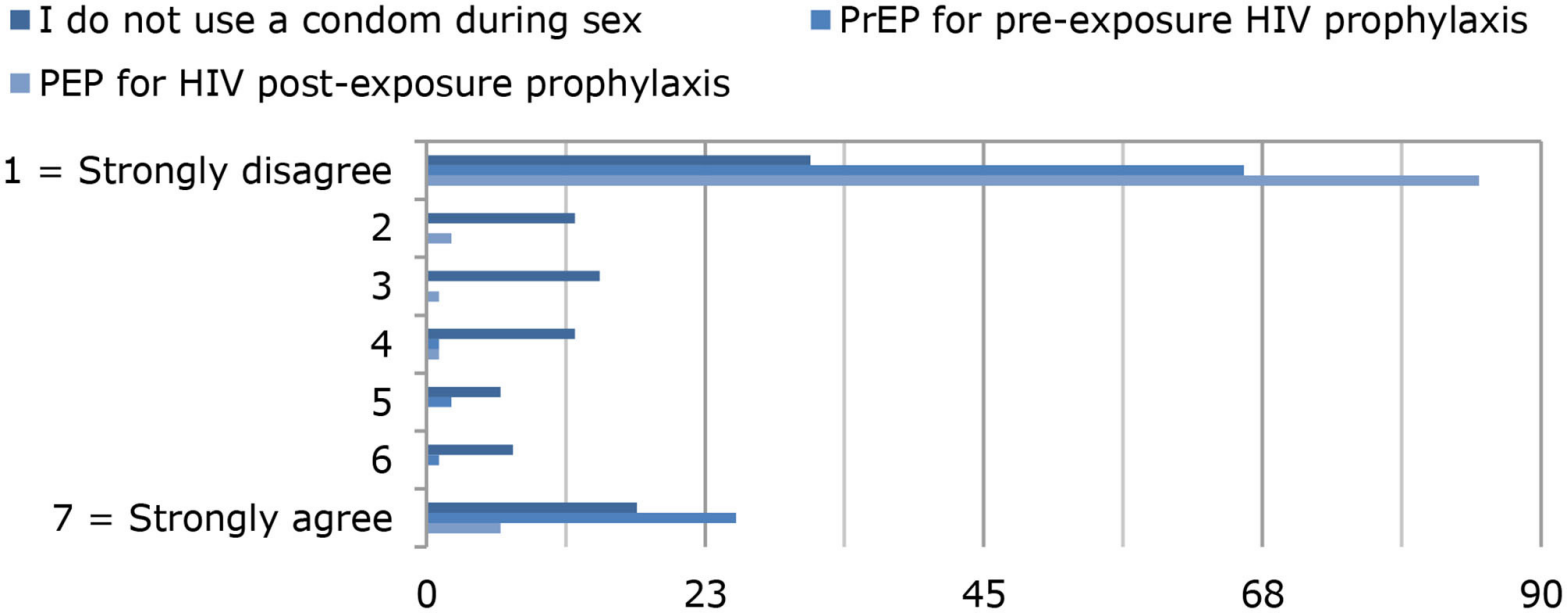
Use of condoms, pre-exposure HIV prophylaxis (PrEP), and post-exposure HIV prophylaxis (PEP).

**Figure 5:**
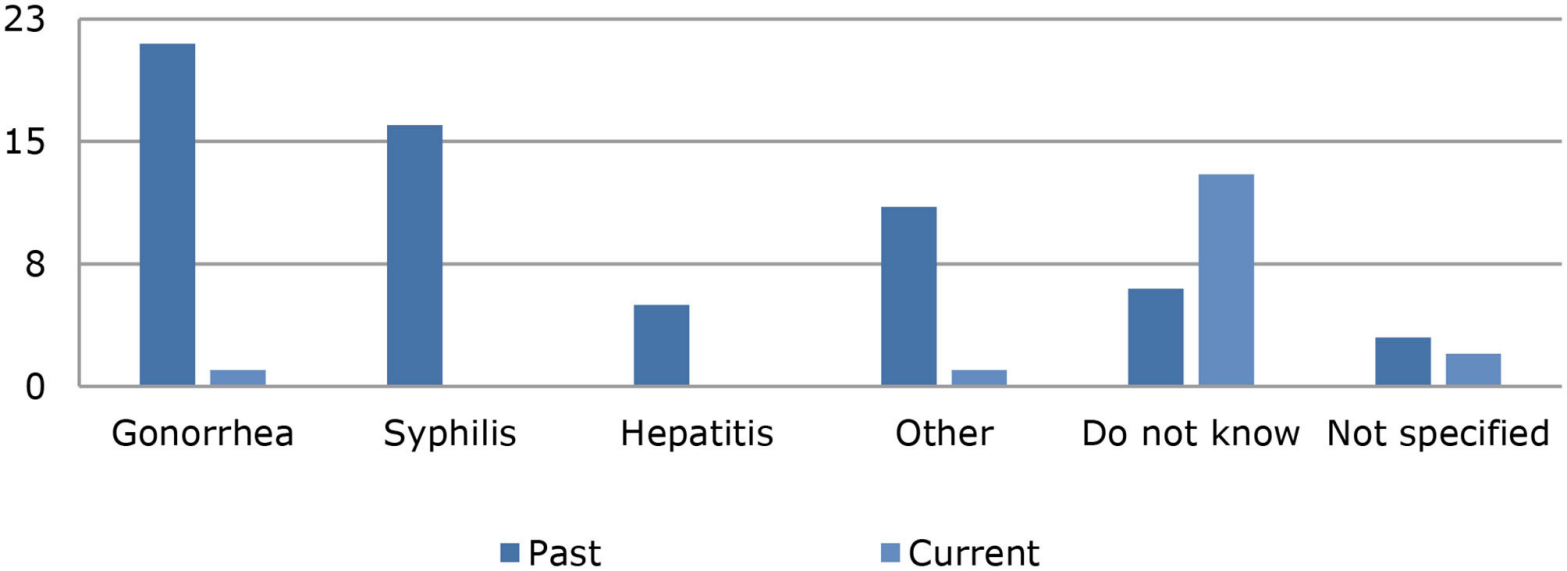
Current and past sexually transmitted diseases among chemsex users.

Regarding sexual behavior with respect to STDs, 31% [mean (SD) = 3.38 (2.26)] of the chemsex users did not use a condom during sex, 66% [mean (SD) = 2.44 (2.24)] did not take pre-exposure drugs for HIV prophylaxis, and 85% [mean (SD) = 1.37 (1.23)] did not take post-exposure drugs for HIV prophylaxis (Figure 4). Figure 4 shows that the majority of the chemsex users did not take any protective measures against STDs: 17% stated that they always use condoms during sex; 31%, that they always use PrEP for HIV prophylaxis; and 7%, that they always use post-exposure HIV prophylaxis. Twenty of the chemsex users stated that they had HIV; information on the prevalence of other STDs is shown in Figure 5. Almost half of the chemsex users (46%) stated that they had been tested for STDs in the past 3 months, and 10% stated that they had never been tested for STDs.

### Need for Advice, Level of Insight, and Desire for Change Among the Chemsex Users

Just over half (58%) of the chemsex users stated that they were not aware of any contact or advice centers focusing on sexual motivated substance use, and 45% stated that they did not know of any advice center in their immediate vicinity. Furthermore, 18% stated that they had obtained information on the topic of sexual motivated drug use from their family physician, on the internet, or from friends. Only a few (3%) said they had contacted an advice center (e.g., for advice on AIDS). One third (30%) had previously received psychiatric or psychotherapeutic treatment; the questionnaire did not ask about the reasons.

Just over half (54%; SD, 1.80; median, 1) of chemsex users stated that they did not consider their sexually motivated substance use to be a problem, and 6% strongly agreed with the statement “I think my chemsex behavior is problematic.” Among the chemsex users, 43% stated that they would not want to do without chemsex in the future (SD, 1.90; median, 2), and 7% strongly agreed with the statement “I would like to do without chemsex in the future.”

## Discussion

In this study, one in three participants stated that they had used illegal drugs in the past 12 months to influence their sexual behavior. The group of chemsex users consisted mainly of homosexual middle-aged men, most of whom were living in a partnership. The majority had an average income and were employed. Most used the 4 chems sporadically, but we did not obtain information on the exact amount of use. The average number of sexual partners, participation in group sex sessions, and lack of protective measures against STDs indicate that participants engaged in risky health behavior. The majority of chemsex users were not aware of contact or advice centers specializing in chemsex, and few of them had previously obtained information or offers of help *via* public or private contact points. Almost half of the chemsex users had moderate to significant insight into the issues associated with their own chemsex behavior, and almost a quarter of chemsex users had no desire to refrain from chemsex in the future. Our findings indicate that overall the chemsex user group has a positive sense of well-being. The predominantly positive well-being can be explained on the one hand by the mostly sporadic substance use and on the other hand by demographic characteristics. Controlled and socially ritualized substance use can reduce undesirable side effects, such as overdoses (41), and therefore has a less negative influence on well-being. Furthermore, this study used the WHO-5, which only examines current well-being and does not record possible past depressive phases. We can also assume that the study sample had social support from a partner and a high socio-economic status through their employment and salary, which in turn has a positive influence on subjectively perceived well-being (42).

Findings on the participants' insight into their own chemsex behavior and the corresponding lack of a desire for change indicate that they had only a low level of suffering. This might be one explanation for the low number of participants who had contacted contact points and advice centers on chemsex. The findings can be further interpreted as showing that some chemsex users are ambivalent about the thought of abstaining from sexual motivated substance use. Noteworthy here is that ambivalence should not be seen as an expression of resistance toward counseling and treatment but as a starting point that can be used to promote motivation and thus to improve one's own health behavior (43). Motivational interviewing may be such a therapeutic starting point (44, 45). We can also assume that chemsex users are a high-risk group with regard to sexual and health behavior and that they are poorly informed about options for advice on chemsex and treatment. This assumption is supported by the findings that chemsex users frequently changed sexual partners and did not always protect themselves against STDs or be tested for them. The low number of contacts with contact and advice centers supports this assumption. Shame and concern about stigma might act as barriers to seeking help and support (46).

The study has some limitations that should be considered when drawing possible conclusions. First, participants were recruited specifically in the LGBTQ community and included an above average number of MSM, especially in the group of chemsex users which can be explained by the snowball sampling. Second, although the WHO-5 has good psychometric properties (39) it records self-reported, subjectively perceived well-being over the past 2 weeks (40). This study did not consider other aspects that may have influenced the well-being and thus mental health of the participants, such as past trauma due to their perceived and lived sexuality; 30% of chemsex users had received psychiatric or psychotherapeutic treatment, so we can assume that they had some kind of psychological stress. Third, this study was an online survey, so it could not control for the conditions of the participants. The participants' responses may therefore have been distorted because they were distracted or influenced by third parties or because they provided incorrect information because they want to give socially desirable responses. Limitations of this study also include the aspect that biomarkers for the use of clinical evaluation substances were not obtained in the process of data collection. Fourth, IP addresses were not saved because of data protection guidelines, so we cannot exclude that one person answered the questionnaire several times. Last, a large proportion of questionnaires were not completed, which may be because the questions about sexual behavior and substance use were perceived as being too intimate or the participants had doubts about data protection.

## Conclusions

Chemsex is becoming more widespread, so we need to understand which interpersonal relationships and influences exist in sexual motivated substance use, taking into account the characteristics of users and their lives. The results of the present study confirmed that chemsex is most common among MSM, although other groups also practice it. In this sample, chemsex users point out that they belong to a special risk group due to lack of protection against sexually transmitted infectious diseases, lack of education about chemsex and little motivation to change. Future research should further investigate the complexity of sexual orientation, sexual behavior, drug use patterns, and motivation for chemsex. A uniform definition of chemsex would improve comparability of studies, and research on this topic would improve the knowledge base. Additional studies on chemsex are urgently needed because chemsex users are known to represent a high-risk group that takes few precautions with regard to STDs and because information on the topic and treatment options are lacking.

## Data Availability Statement

The raw data supporting the conclusions of this article will be made available by the authors, without undue reservation.

## Ethics Statement

The studies involving human participants were reviewed and approved by the ethical committee by the LMU and was registered by the number: 18-819KB. The patients/participants provided their written informed consent to participate in this study.

## Author Contributions

CR: conceptualization, methodology, software, validation, formal analysis, investigation, data curation, writing—original draft, visualization, and project administration. MG: conceptualization, methodology, writing—review, editing, and investigation. MS: investigation, visualization, writing—review, and editing. JS, SG, and AR: writing—review and editing. EL: methodology, supervision, writing—review, and editing. TR: resources, writing—review, editing, and supervision. All authors contributed to the article and approved the submitted version.

## Conflict of Interest

The authors declare that the research was conducted in the absence of any commercial or financial relationships that could be construed as a potential conflict of interest.
