# Supplementary material for: We Have a Lot to Do: Lack of Sexual Protection and Information—Results of the German-Language Online Survey “Let's Talk About Chemsex”
Source: Front Psychiatry. 2021 May 31;12:690242. doi: 10.3389/fpsyt.2021.690242 (PMC8200571; doi:10.3389/fpsyt.2021.690242)
Supplement: Supplementary file 1 [file Table_1.docx]

**A) Use of psychoactive substances in the group of chemsex users (n = 123)**

| **Substance** | **Frequency of use** | | | | | | |
| --- | --- | --- | --- | --- | --- | --- | --- |
|  | **Daily** | **Several times a week** | **Approx. once a week** | **Approx. once a month** | **Approx. once every 3 months** | **Never** | **Not specified** |
| **Alcohol** | 6 | 35 | 27 | 29 | 16 | 8 | 2 |
| **Cannabis** | 6 | 18 | 8 | 8 | 30 | 48 | 5 |
| **Synthetic Cannabinoids** | - | 2 | 1 | 3 | 3 | 106 | 8 |
| **Ketamine** | - | - | 4 | 8 | 17 | 87 | 7 |
| **Amphetamine** | 1 | 3 | 6 | 12 | 24 | 72 | 5 |
| **Methamphetamine** | 1 | 3 | 1 | 7 | 11 | 93 | 5 |
| **MDMA** | 1 | 2 | 2 | 15 | 25 | 72 | 6 |
| **Mephedrone** | - | 1 | 2 | 10 | 11 | 93 | 6 |
| **Cocaine** | - | 3 | 6 | 9 | 26 | 73 | 5 |
| **Crack** | - | - | - | - | 1 | 112 | 10 |
| **Heroin** | - | - | - | - | 1 | 113 | 9 |
| **LSD** | - | - | 1 | - | 3 | 112 | 7 |
| **Inhalants**  (for example Sniffing substances like glue) | - | 1 | 4 | 2 | 7 | 103 | 6 |
| **Biogenic drugs**  (for example Datura, Angel´s Trumpet) | - | 1 | - | - | 4 | 111 | 7 |
| **GHB/GBL** | - | 5 | 5 | 11 | 27 | 71 | 4 |
| **Poppers** | 3 | 33 | 34 | 16 | 18 | 17 | 2 |
| **Prescription painkillers without a physician’s prescription** | 1 | 3 | 1 | - | 8 | 104 | 6 |
| **Prescription painkillers with a physician’s prescription** | - | - | 1 | 2 | 10 | 103 | 6 |
| **Benzodiazepines** | 1 | - | - | 3 | 10 | 102 | 7 |
| **Barbiturates** | - | 1 | - | - | 4 | 110 | 8 |
| **Other prescription drugs without a physician’s prescription** | - | - | 1 | 1 | 7 | 107 | 7 |
| **Other prescription drugs without a physician’s prescription** | 2 | 1 | - | - | 3 | 110 | 7 |
| **Nicotine** | 44 | 8 | 4 | 3 | 10 | 49 | 5 |
