# Supplementary material for: We Have a Lot to Do: Lack of Sexual Protection and Information—Results of the German-Language Online Survey “Let's Talk About Chemsex”
Source: Front Psychiatry. 2021 May 31;12:690242. doi: 10.3389/fpsyt.2021.690242 (PMC8200571; doi:10.3389/fpsyt.2021.690242)
Supplement: Supplementary file 2 [file Table_2.docx]

**B) Scores for sexual and sexual health-related behavior in 402 participants in an online survey (not all data shown). Items were rated on a 7-point Likert scale ranging from 1 (strongly disagree) to 7 (strongly agree)**

| **Item** | **Chemsex users**  **(n = 123)**  **Mean (SD)** | **Non-users**  **(n = 279)**  **Mean (SD)** |
| --- | --- | --- |
| Number of sexual partners | 24.59 (33.49)  Median = 12 | 6.61 (17.11)  Median = 2 |
| In the past 12 months, I had sex with more than 1 person at the same time. | 4.71 (2.70) | 2.27 (2.37) |
| I meet my sexual contacts spontaneously in bars and do not prearrange the meetings. | 3.07 (2.32) | 1.92 (1.68) |
| I use web portals to arrange to meet someone for sex. | 5.70 (1.95) | 2.75 (2.39) |
| I go to private sex parties. | 3.16 (2.41) | 1.57 (1.54) |
| I go to known bars to find sexual partners (eg, darkrooms, saunas, swinger clubs, private clubs, etc.). | 3.46 (2.49) | 1.84 (1.81) |
| I like having sex with men. | 6.69 (1.10) | 5.57 (2.27) |
| I like having sex with women. | 2.27 (2.14) | 3.07 (2.49) |
| I like having one night stands. | 4.94 (2.06) | 2.98 (2.18) |
| I like having sex in a relationship. | 5.63 (1.83) | 6.30 (1.33) |
| I like having sex outside a relationship. | 5.10 (2.12) | 3.36 (2.34) |
| I like having sex with several people at the same time. | 5.20 (1.94) | 2.77 (2.22) |
| I like having sex with prostitutes. | 1.83 (1.65) | 1.25 (0.89) |
| I use condoms when I have sex. | 3.38 (2.26) | 4.46 (2.42) |
| I use HIV pre-exposure prophylaxis before I have sex. | 2.44 (2.24) | 1.22 (1.00) |
| I use HIV post-exposure prophylaxis before I have sex. | 1.37 (1.23) | 1.12 (0.74) |
| When having sex in a relationship, I do not use contraceptives to protect myself against sexually transmitted diseases. | 4.80 (3.04) | 4.46 (2.61) |
| When having sex outside a relationship, I do not use contraceptives to protect myself against sexually transmitted diseases. | 2.95 (2.42) | 1.64 (1.48) |
